# Fine-Scale Genetic Structure of *Curculio chinensis* (Coleoptera: Curculionidae) Based on Mitochondrial *COI*: The Role of Host Specificity and Spatial Distance

**DOI:** 10.3390/insects15020116

**Published:** 2024-02-06

**Authors:** Li Zhang, Fuping Wang, Jiaxi Wu, Sicheng Ye, Ye Xu, Yanan Liu

**Affiliations:** 1 Institute of Jiangxi Oil-Tea Camellia, Jiujiang University, Jiujiang 332005, China; fpw616@126.com (F.W.); wjx15979259429@163.com (J.W.); yesicheng520@163.com (S.Y.); liuyanan81211@163.com (Y.L.); 2 School of Agricultural Science, Jiangxi Agricultural University, Nanchang 330045, China; yexu@jxau.edu.cn

**Keywords:** *Curculio chinensis*, fruit-boring pest, genetic structure, *COI*, oil-tea *Camellia*

## Abstract

**Simple Summary:**

*Curculio chinensis* is a phytophagous pest that feeds on oil-tea *Camellia* in South and Southwest China. This pest is highly dependent on its hosts and habitats. The genetic basis of this pest in various hosts, which could enrich our understanding of whether host-specificity existed and how the population is structured, is poorly explored. This study aimed to evaluate the genetic diversity, genetic differentiation, and phylogenetic structure of *C. chinensis* in 2 major host species, *Camellia meiocarpa* Hu and *Camellia oleifera* Abel, using 1083 mitochondrial *COI*. Structural differentiation was observed among populations in monoculture plantations of *Camellia meiocarpa* and *Camellia oleifera*. The mean genetic distance between Haplogroup 1 and Haplogroup 2 was significantly lower than that between *C. chinensis* and its related species. Two haplogroups have recently undergone a demographic expansion, and a significant asymmetrical effective migration was observed from *C. chinensis* populations in *Cam. meiocarpa* to populations in *Cam. oleifera* in comparison to migrating back, which is likely due to the increased cultivation of oil-tea Camellia in Jiangxi. Our findings can serve as a guide for future genomic research to improve prediction and facilitate control strategies for *C. chinensis*.

**Abstract:**

The Camellia weevil, *Curculio chinensis* (Chevrolat, 1978), is a dominant oligophagous pest that bores into the fruit of oil-tea *Camellia*. Genetic differentiation among populations in various hosts can easily occur, which hinders research on pest management. In this study, the genetic structure, genetic diversity, and phylogenetic structure of local *C. chinensis* populations were examined using 147 individuals (from 6 localities in Jiangxi), based on 2 mitochondrial *COI* markers. Results indicated that the *C. chinensis* population in Jiangxi exhibits a high haplotype diversity, especially for the populations from *Cam. meiocarpa* plantations. Structural differentiation was observed between Haplogroup 1 (73 individuals from Ganzhou, Jian, and Pingxiang) in the monoculture plantations of *Cam. meiocarpa* and Haplogroup 2 (75 individuals from Pingxiang and Jiujiang) in *Cam. oleifera*. Two haplogroups have recently undergone a demographic expansion, and Haplogroup 1 has shown a higher number of effective migrants than Haplogroup 2. This suggests that *C. chinensis* has been spreading from *Cam. meiocarpa* plantations to other oil-tea *Camellia*, such as *Cam. oleifera*. The increased cultivation of oil-tea *Camellia* in Jiangxi has contributed to a unique genetic structure within the *C. chinensis* population. This has, in turn, expanded the distribution of *C. chinensis* and increased migration between populations.

## 1. Introduction

*Curculio chinensis* (Chevrolat, 1978) (Coleoptera: Curculionidae), one of the dangerous forest pests in China, was first reported in the 1960s. It is now distributed in oil-tea *Camellia* plantations in South and Southwest China, but it has not been found abroad [1]. *C. chinensis* predominantly damages the fruits and seeds of *Camellia* species [2]. Young tea leaves and shoots can be damaged by adults’ piercing-sucking, which can then easily lead to infection by anthracnose. The eggs and larvae were deposited in fruits of *Camellia* species, leading to spoiled seeds or even complete fruit failure [3,4,5].

China is the origin and distribution center of *Camellia* spp. The vast majority of oil-tea *Camellia,* a unique woody-oil species, is located in China [6]. *C. chinensis* is a notorious oligophagous pest with weak flying ability in the primary oil-tea *Camellia* plantation in China. The investigation of the population showed that the occurrence of *C. chinensis* is closely related to the host species and the climate of plantations [7,8]. *C. chinensis* and *C.* spp. have shown significant variation related to host isolation (*Cam. oleifera* and *Cam. sinensis*) using mitochondrial *COI*. For *C. chinensis,* the tea saponin content varies among host species and affects the composition of gut microbiota. The current study focuses on the adaptation of gut bacteria to the phytochemical resistance of oil-tea *Camellia* [9,10]. For other species of Coleoptera with limited flight ability, natural geographical factors and host plants often play crucial roles in influencing the genetic structure and gene flow among populations. *C. camellia*, a closely related species to *C. chinensis*, is influenced not only by geographical isolation but also by host plants [11], and the migration pattern of *C. camellia* imposed significant effects on the geographic configuration of the coevolution level between *C. camellia* and its host, *Cam. Japonica* [12,13]. Various levels of geographic differentiation and host specificity have been detected among interspecific and intraspecific species of *Camellia* using mitochondrial genes [14,15]. Therefore, in addition to gut bacteria adaptation, the genetic structure characteristics and gene flow patterns of *C. chinensis* are speculated to vary due to diverse host species and geographical isolation. This makes *C. chinensis* an ideal subject for revealing adaptive evolution.

Mitochondrial genes, such as *COI*, have the advantages of stable composition and conservative gene arrangement and have been extensively utilized in population genetics studies of Curculionidae [11,14,15]. Although mitochondrial genes are maternally inherited, their small effective size, interspecific diversity, and relatively high mutation rates make them especially valuable for providing crucial insights into population dynamics, predicting potential migration events, and gaining a better understanding of the evolutionary potential related to hosts and habitats. But shorter mitochondrial gene sequences often lack sufficient variable sites to accurately resolve genetic diversity and population differentiation. For example, the results based on mitochondrial *COI* (544 bp) revealed a unique haplotype in the Jiangxi population, which was significantly different from the other populations in China [16,17]. However, mitochondrial ATP synthase-based results showed higher diversity and more private haplotypes in the Jiangxi population compared to the other populations [18]. On the other hand, the aforementioned findings also suggest that there may be local adaptive differentiation in the Jiangxi population.

Jiangxi Province is one of the major cultivation regions for oil-tea Camellia in China. The various *Camellia* species in Jiangxi provide diverse habitats for the occurrence of *C. chinensis*, making Jiangxi Province an ideal area to study the local adaptability of this pest. In the present study, our main objective was to test the host specificity of *C. chinensis* and examine its population dynamics while taking into account the genetic variation of this pest collected from two major species, *Camellia meiocarpa* Hu and *Camellia oleifera* Abel, within Jiangxi plantations. First, the population diversity, genetic differentiation, and phylogeographical structure of *C. chinensis* were assessed using variations in mitochondrial *COI* sequences. Subsequently, population dynamics were estimated from recurrent migration between genetic haplogroups and the demographic history of populations. The correlation between genetic differentiation and host plants, as well as geographical isolation, was evaluated using an analysis of molecular variance and a matrix correspondence test. This will infer the adaptive differentiation of *C. chinensis* to hosts and geographical factors and will enable better prediction and management of *C. chinensis* in Jiangxi.

## 2. Materials and Methods

### 2.1. Sample Collection

**Figure 1:**
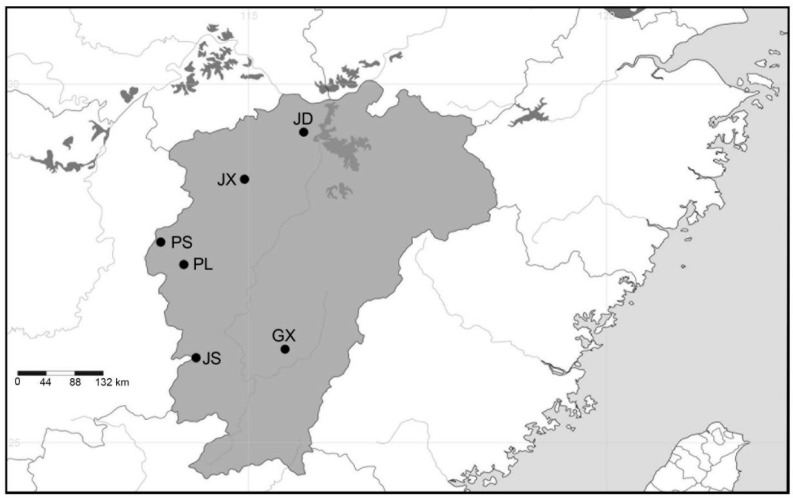
The distribution of six *Curculio chinensis* populations in Jiangxi, China. Population codes are listed in Table 1. SimpleMappr was used to produce a distribution map based on the geographical coordinates in Table 1. URL: http://www.simplemappr.net/#tabs=0 (accessed on 25 December 2023).

During 2022 and 2023, specimens of *C. chinensis* were selectively collected from six localities in Jiangxi (Figure 1) using a Z-sampling method (six plots from each locality). These localities were infested by this weevil over the years and covered species-distributed regions, including *Cam. meiocarpa* plantations, *Cam. oleifera* plantations, and areas with a mix of *Cam. meiocarpa*/*Cam. oleifera* plantations (Table 1; Figure 1). A total of 1000 specimens were preserved in absolute alcohol at −20 °C until they were identified and used for DNA extraction. They are now deposited at the Institute of Jiangxi Oil-tea Camellia, Jiujiang University, Jiujiang, China. A total of 1 adult (identified using morphological characters [1] and sequencing firstly in populations) and 21–24 mature larvae were chosen for 6 populations.

### 2.2. DNA Extraction, Optimization of PCR Amplification

Genomic DNA was extracted from a leg of an adult or muscle tissue of larvae using the EsayPure^®^ Genomic DNA Kit (TransGen Biotech, Beijing, China). The remaining specimens were deposited. DNA concentration was measured using an ND-1000 spectrophotometer (Bio-Rad, Hercules, CA, USA) and diluted to 20 ng/μL with ddH_2_O.

To obtain adequate variable sites for genetic diversity and population differentiation resolution, mitochondrial *COI* was amplified using two pairs of primers (10 µM): LCO1490(F): GGTCAAC AAATCATAAAGATATTGG, HCO2198(R): TAAACTTCAGGGTGACCAAAAAATCA [16] and COS1751C(F): GGAGCTCCTGATATAGCTTTYCC, COAZ1(R): TGAATAAT GGGAATCATTGAAC [11]. A final volume of 25 µL contained 2 µL genomic DNA, 2 μL dNTP Mix, 2.5 μL 10 × PCR Buffer, 0.25 μL Taq DNA polymerase (TaKaRa, Dalian, China), 1 μL each of forward and reverse primers, and was supplemented with 25 μL of ddH_2_O. The polymerase chain reaction (PCR) program was performed using the following steps: an initial denaturation step of 5 min at 95 °C; 35 cycles of denaturation at 95 °C for 30 s; annealing at 50 °C for 40 s, (annealing temperature was determined by results of electrophoresis); extension at 72 °C for 30 s; and a final extension at 72 °C for 5 min. After being examined using 1.5% agarose gel electrophoresis, the amplification products were sequenced using the ABI 3730 automated sequencer (Applied Biosystems, Foster City, CA, USA) at Beijing Tsingke Biotech Company (Beijing, China). *Curculio davidi* Fairmaire was selected as the outgroup.

### 2.3. Sequencing and Population Genetic Structure Analysis

#### 2.3.1. Genetic Diversity Analyses

DNA sequences were derived from 147 individuals selected from 6 locations representing 6 populations. A total of 2 fragments of 544 bp and 825 bp were obtained from each sample using 2 pairs of primers. The raw sequences were then proofread using Geneious Primer v. 11.0.14.1 [19]. Sequences of target genes were confirmed by aligning the resulting sequences to the mitochondrial genome MZ417388 [20] in the National Center for Biotechnology Information (NCBI) database using the BLAST tool. The aligned sequences were then joined together using MEGA v. 7.0 [21]. Mitochondrial *COI* sequences from 147 samples, each 1083 bp long, were used for genetic diversity analyses. The number of haplotypes (Nh), haplotype diversity (Hd), nucleotide diversity (π), the number of polymorphic sites (S), and the mean number of nucleotide difference (k) were estimated by DnaSP v. 6.12.03 [22].

#### 2.3.2. Phylogenetic Analyses and Genetic Structure

Phylogenetic trees were reconstructed using 147 mitochondrial *COI* sequences and 5 sequences from GenBank (MF409663, MF409669, MF409675, MF409681, and MF409682). Maximum likelihood (ML) and Bayesian inference (BI) were performed using IQ-TREE and MrBayes in PhyloSuite v.1.2.3 [23,24] after finding the GTR+ F +G4 model based on the Akaike information criterion (AIC) in the ModelFinder. Two sequences (OR976214, OR976215) of *C. davidi* were set as the outgroup. A total of 5000 ultrafast bootstraps and 1000 replicates for the SH-aLRT branch test were run in ML [25]. MrBayes uses Markov chain Monte Carlo (MCMC) to perform Bayesian inference of phylogeny [26]. A total of 2 separate analyses, 4 MCMC chains, 2 × 10^6^ generations (the removal of the first 25% of samples), and 1000 sampling statistics were set for running until the average standard deviations of split frequencies were below 0.01 and the effective sample size (ESS) was above 100 [27]. The tree was visualized and edited with an online tool called the Interactive Tree of Life (iTOL) v. 5 [28].

Haplotype networks were analyzed and edited using Network v. 10 to infer relationships between haplotypes from 147 samples. An admixture model was chosen in the Bayesian inference-based software STRUCTURE v. 2.3.4 [29,30] to detect clusters of multi-site haplotypes in populations using correlated allele frequencies. Inferred clusters (K) 1 to 6 were set with 20 independent runs of each and 1 × 10^6^ (MCMC) repetitions (a 100,000 repetition burn-in period in each run). CLUMPP v1.1.2 [31] and DISTRUCT v. 1.1 [32] were used to permute the cluster labels across runs and display the genetic structure results after determining the most likely number of genetic clusters (K) based on the result of the ad hoc statistic (∆K) using STRUCTURE HARVESTER [33,34]. Sequence divergences among haplogroups and other *COI* sequences from 11 mitogenomes (MT560591, MK654677, KX087269, MG728095, NC045101, NC027577, KX087330, MT232762, MW023069, NC022680, NC051548) in the same family downloaded from GenBank were calculated with MEGA v. 7.0 [21] using Kimura two-parameter distances.

#### 2.3.3. Isolation by Hosts and Distance Analysis

The analysis of molecular variance (AMOVA) was performed to partition genetic variations among haplogroups and within haplogroups, which were divided based on the results of haplotype relationship analyses in Arlequin version 3.5.2 [35] with 10,000 permutations, as well as the fixation indices. The genetic differentiation between populations of different hosts in the same sample site and AMOVA among haplogroups in different sample sites were also conducted to infer the effects of the host.

Within Jiangxi plantations, the anticipated high levels of gene flow at a small spatial scale should somewhat restrict the geographic differentiation of the weevil trait. We thus predicted that the difference in genetic distances of populations would not be corrected with increasing geographic distance between weevil populations if the extent of gene flow affected the degrees of local adaptation. Mantel tests, in which the effects of geographic distance between localities were controlled, were conducted to examine the correlation between geographic distance and interpopulation genetic distance using IBDWS v. 3.23 [36,37] The pairs of geographic distance/genetic distance between populations were generated using Geographic Distance Matrix Generator v. 1.2.3 and MEGA v. 7.0.

#### 2.3.4. Population Dynamics

Neutrality tests (Tajima’s D and Fu’s Fs statistics) of 6 populations were calculated to test for evidence of recent population expansion using DnaSP v. 6.12.03 [38,39]. To infer the gene flow between haplogroups, Bayesian inference of population genetic parameters was conducted using the program Migrate-n 3.1.6. The DNA sequence model and the full model were used to estimate the migration rate (*M*) and the mutation-scaled population size (*θ*) [40]. After the initial run with *F*_ST_, *θ* and *M* were used for the remaining three runs. One hundred million MCMC steps were taken, with the first 2 × 10^6^ steps discarded, and static heating schemes with 4 chains were sampled to estimate *θ* and *M* in the Bayesian search strategy. The effective number of migrants of each population per generation (Nem) can be calculated as 4*θM*, and the effective population size (N) of each population can be calibrated by the mutation rate (N = *θ/2μ*; *μ*, mutation rate per site per generation is assumed to be 1.8 × 10^−8^ [13]). N and Nem can reveal the effective population size and population interactions of *C. chinensis*, which can help us recognize the focal population and immigration of this hidden pest for monitoring and control.

## 3. Results

### 3.1. Genetic Diversity

A total of 36 haplotypes were identified from 147 *COI* sequences in six populations of *C. chinensis* (OR976178-OR976213). High genetic diversity was detected in Jiangxi populations. The number of haplotypes in various populations ranged from 4 to 9, with haplotype diversity ranging from 0.545 to 0.777 and nucleotide diversity ranging from 0.00075 to 0.02496. The number of polymorphic sites (S) and the mean number of nucleotide difference (k) were 0.81 to 27.033 and 3 to 75, respectively (Table 1). Five common haplotypes were found. Populations in *Cam. meiocarpa* plantations and *Cam. oleifera* plantations have the most common haplotypes, H8 and H15, respectively (Table A1). There were clear differences in the number of polymorphic sites (S) and the mean number of nucleotide differences (k) between *C. chinensis* populations from *Cam. oleifera* plantations and *Cam. meiocarpa* plantations. For populations in different plantations, the highest S (18) and k (1.8) were found in PS populations, while the lowest S (3) and k (0.633) were discovered in the JX population (Table 1).

### 3.2. Haplotype Relationship and Genetic Differentiation Analyses

**Figure 2:**
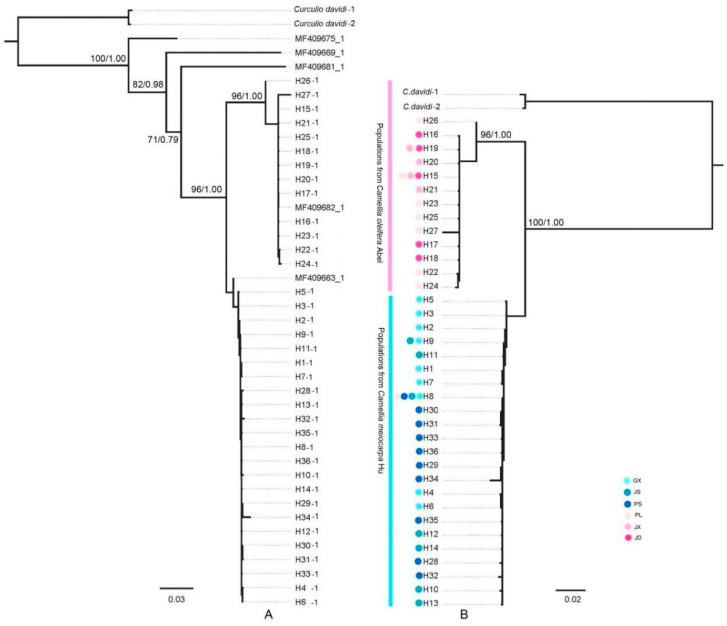
The phylogenetic tree of *C. chinensis* populations using maximum likelihood (ML) and Bayesian inference (BI). (**A**). MF409663, MF409669, MF409675, MF409681, and MF409682 are downloaded haplotypes. H1-1–H36-1 (544 bp) are aligned with the downloaded haplotypes. (**B**). H1–H36 (1083 bp) are haplotypes from Jiangxi populations. The bootstrap values of ML and the posterior probability of BI are given (>90/0.9).

Thirty-six haplotypes were used for the phylogenetic analysis. ML and Bayesian results showed that haplotypes of *C. chinensis* populations were separated into two haplogroups with high support values (Figure 2). Haplogroup 1 mainly includes 23 haplotypes from GX, JS, and PS populations; the other haplotypes from JD, PL, and JX populations clustered into Haplogroup 2. Haplogroup 1 clustered with MF409663 in Clade 1, while Haplogroup 2 clustered with MF409682 in Clade 2 based on 544 bp in a previous study [17] (Figure 2A). The haplotypes of populations in *Cam. oleifera* plantations clustered closely, as well as the haplotypes of populations in *Cam. meiocarpa* plantations. The common haplotypes H8 and H15 were detected in the PL population (Figure 2B).

**Figure 3:**
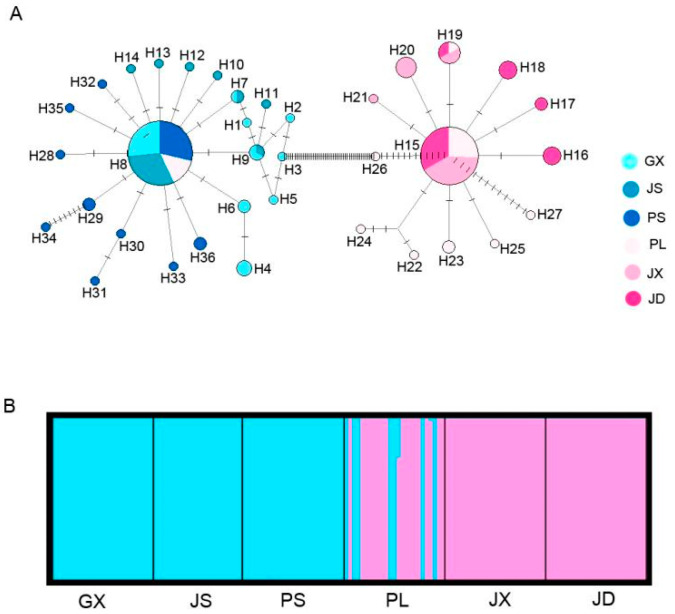
Haplotype network and STRUCTURE analysis of *C. chinensis* populations based on concatenated mitochondrial *COI*. (**A**). Each circle represents a haplotype, and the sizes indicate the number of individuals. (**B**). The proportion of populations from two clusters inferred by STRUCTURE analysis. An individual is represented by a vertical bar.

**Figure A1:**
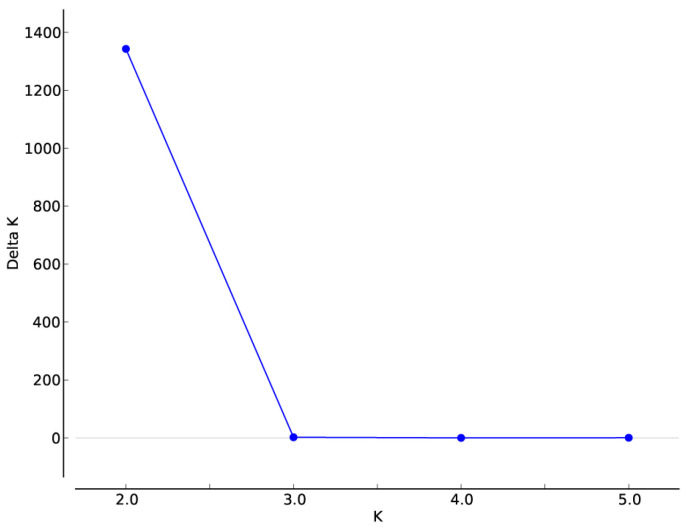
Estimated number of genetic groups obtained with structure analysis for K ranging from one to six using mitochondrial *COI* for six populations. The most likely number of genetic clusters (K) was two.

The results of haplotype networks and STRUCTURE corresponded to two haplogroups in the phylogenetic tree (Figure 3; Figure A1). H8 and H15 were common haplotypes shared by 49 and 39 individuals in different plantations. The others were private in one population, except for H7 in GX and JS, and H19 in JD, JX, and PL. The GX, JS, and PS populations have a similar genetic composition in Haplogroup 1, while the genetic composition of JD is similar to JX in Haplogroup 2. PL has the most complex genetic composition in both Haplogroup 1 and Haplogroup 2.

### 3.3. Isolation by Hosts and Distance Analysis

The genetic distances within the haplogroups of *C. chinensis* ranged from 0.0011 to 0.018, with a mean of 0.0013 for Haplogroup 1 and 0.0122 for Haplogroup 2. The genetic distances between Haplogroup 1 and Haplogroup 2 ranged from 0.0415 to 0.0593, and those between species within *Curculio* ranged from 0.1279 to 0.1992. The genetic distances between *C. chinensis* and related species within Curculionidae ranged from 0.1710 to 0.2392, with a mean of 0.2045 (Table A2).

**Figure A2:**
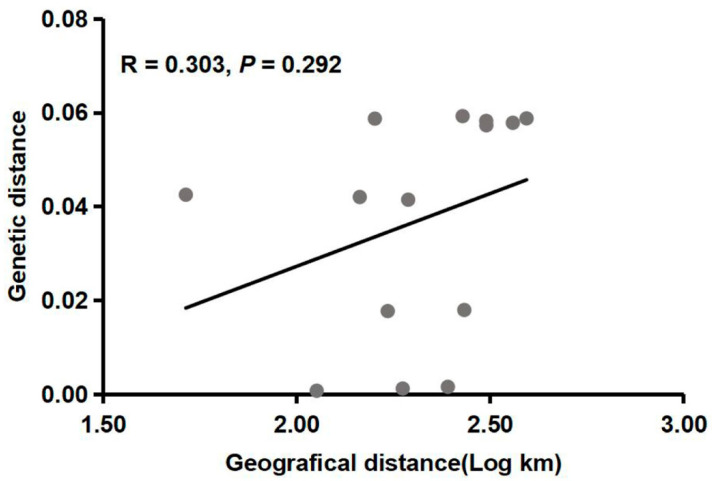
Scatter plots of the genetic distance isolation by geographical distance among six *Curculio chinensis* populations in Jiangxi based on mitochondrial *COI*.

Mantel tests showed no correlation between geographic distance and genetic distance (r = 0.303, *p* = 0.292) (Figure A2). The *C. chinensis* population was genetically differentiated among haplogroups (FCT = 0.870, *p* < 0.0001), but a low-level genetic differentiation was found between populations within haplogroups (FSC = 0.226, *p* < 0.0001 (Table 2)). The AMOVA indicated that the majority of the genetic variance was among populations of different hosts (88.32%), rather than within populations (11.90%).

### 3.4. Population Dynamics

The results of Tajima’s *D* and Fu’s Fs indicate a recent expansion for JS, PS, JD, and JX populations (Table 1). Significant population expansion was supported by both Tajima’s *D* and Fu’s *Fs* in the JS and PS populations (Tajima’s *D* < 0, *p* < 0.05; Fu’s *Fs* < 0, *p* < 0.05). The JD and JX populations have also undergone a population expansion, but the expansion was not significant (Tajima’s *D* < 0, *P* > 0.05; Fu’s *Fs* < 0, *p* > 0.05). The significantly negative values of Tajima’s *D* and Fu’s *Fs* suggest a recent demographic expansion for Haplogroup 1 (Tajima’s *D* = −2.417, *p* < 0.01; Fu’s *Fs* = −22.941, *p* < 0.01). Populations in Haplogroup 2 showed evidence of a recent demographic expansion, as indicated by the negative value of Fu’s Fs (Fu’s Fs: −0.950, *p* > 0.100).

## 4. Discussion

### 4.1. Genetic Diversity of C. chinensis in Different Hosts

*C. chinensis* populations in Jiangxi have substantially high genetic diversity. The results were different from those reported in the Jiangxi population [17] but similar to the results in *C. camellia* [11]. This can be attributed to multiple mutation sites from 6 populations in different hosts based on 1083 bp mitochondrial sequences (70% complete mitochondrial *COI* [20]) in this study, while 1 conserved site from only 1 population in a natural and isolated *Cam. Oleifera* plantation near the Wuyi Mountains based on 544 bp mitochondrial sequences in the previous study [17]. The genetic diversity (S and k) of GX, JS, and PS populations in *Cam. oleifera* plantations was lower than that of JX and JD populations in *Cam. meiocarpa* plantations. Additionally, significant differences in S and k were detected in PL and PS populations, which have similar geographical locations (near the Luoxiao Mountains) but different hosts. Unique private haplotypes were found in populations collected from *Cam. oleifera* plantations (11, 30.5% of all haplotypes) and *Cam. meiocarpa* plantations (20, 55.5% of all haplotypes) (Table A1; Table 1). This suggests significant genetic variation among samples from different hosts.

### 4.2. Population Genetic Structure and Nucleotide Divergences

Two haplogroups were detected in samples from Jiangxi, which were from *Cam. meiocarpa plantations* and *Cam. oleifera* plantations, respectively. The results of haplotype phylogenetic relationships, networks, and STRUCTURE can elucidate the relationship between two haplogroups. In this study, 36 haplotypes were observed, but only H8 (PL population) was shared by two haplogroups. The PL population from the plantation includes *Cam. Oleifera,* and *Cam. meiocarpa* also has the same genetic components as the two haplogroups from *Cam. oleifera* and *Cam. meiocarpa* plantations, respectively (Figure 2B). The AMOVA analysis revealed a high level of genetic differentiation among the haplogroups with two hosts and populations in different haplogroups.

**Figure 4:**
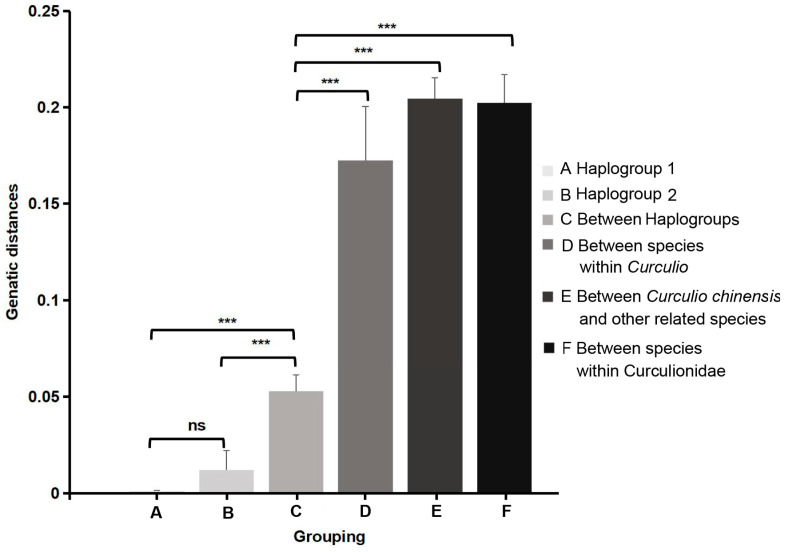
Comparison of genetic distances between different groupings. A: the mean genetic distances of GX, JS, and PS *C. chinensis* populations; B: the mean genetic distances of JD, PL, and JX *C. chinensis* populations; C: the mean genetic distances between Haplogroup 1 and Haplogroup 2; D: the mean genetic distances within *Curculio*; E: the mean genetic distances between *C. chinensis* and its related species; and F: the mean genetic distances between species within Curculionidae, except for *C. chinensis.* Genetic distances are shown in Table A2. *** and ns denote significant difference and no significant difference, respectively.

The mean genetic distances within haplogroups did not exhibit any significant difference (A/B: *t*-test: t = 1.911, d.f. = 4, *p* = 0.196), which was notably lower than the distances between haplogroups (with a mean of 0.0529) (C/A: *t*-test: t = 18.823, d.f. = 10, *p* < 0.001; C/B: *t*-test: t = 7.137, d.f. = 10, *p* < 0.001). The mean genetic distance between Haplogroup 1 and Haplogroup 2 was significantly less than that between *C. chinensis* and its related species (C/D: *t*-test: t = −9.387, d.f. = 12, *p* < 0.001; C/E: *t*-test: t = −36.402, d.f. = 25, *p* < 0.001) (Figure 4) [41]. The genetic distance between the two haplogroups of *C. chinensis* was greater than the intraspecific distance of other species in Jiangxi. This is similar to the intraspecific genetic variation found in one-quarter of the species from BOLD (>0.03) [42,43]. The above situation suggests a clear differentiation between the two haplogroups, which may be related to host shifts.

### 4.3. Fine-Scale Population Dynamics and Potential Effects on Genetic Differentiation

Host shifts can lead to directional gene flow and result in genetic differentiation or even speciation. So, host plant specialization is a critical mechanism for the diversification of phytophagous insects. Insect species with poor migration ability often exhibit genetic differentiation among populations that feed on different host species. This is exemplified by the genetic divergence observed between *C. chinensis* populations in this study. Haplotype phylogenetic relationships based on 1083 bp *COI* showed that populations collected from the same host were clustered together. This may be associated with a potential microbial contribution to the chemical adaptability of tea saponin of *Camellia* species [9,10]. Some previous studies have also indicated host-associated divergence in *Curculio* species [13,15,44].

Our Bayesian estimation of population genetic parameters revealed that GX, JS, and PS *C. chinensis* populations (N = 5.18 × 10^5^, Nem1→2 = 4.96), which were clustered in Haplogroup 1, were potential source populations, On the other hand, JD, PL, and JX *C. chinensis* populations in Haplogroup 2 (N = 3.21 × 10^5^, Nem2→1 = 1.00) were identified as sink populations (Table A3). Significant asymmetrical effective migrants (Nem) between Haplogroup 1 and Haplogroup 2 were discovered through non-overlapping 95% confidence intervals. These close phylogenetic relationships and asymmetrical effective migration suggest that *C. chinensis* populations might have shifted their range from *Cam. meiocarpa* plantations to *Cam. oleifera* plantations. No significant geographical differentiation was found between six populations with a similar subtropical monsoon climate (no significant difference in annual mean temperature (19.17–18.35)), but a complex genetic component in the PL population from *Cam. oleifera*/*Cam. meiocarpa* plantations and Bayesian estimation of population-effective migrants suggest that genetic differentiation between local *C. chinensis* populations has been caused by artificial cultivation, transformation, and host adaptability rather than physical barriers.

Genetic migration of many parasitic pests was affected by human plant breeding and commerce. Previous research has reported the effects of human activities on population divergence between *Curculio* beetles [45,46,47]. As the cultivation area of *Cam. Oleifera* is increasing, *C. chinensis* is occurring across wider areas. *Cam. meiocarpa* and *Cam. oleifera*, the two most widely distributed species in Jiangxi, were promoted for planting from the 1950s and the 1990s, respectively. Many plantations that included these two species were retained during the low-yield transformation of oil-tea *Camellia*. *C. chinensis* can be transferred by the seeds during the larval stage and dispersed across different trees in the same plantations at the adult stage. So, the recent demographic expansion was found in JS, PS, JD, and JX populations. Our results also showed that there were more migrants from Haplogroup 1 to Haplogroup 2 than from Haplogroup 2 to Haplogroup 1, and Haplogroup 1 has a closer relationship with MF409663 in Clade 1 from five other provinces in China than Haplogroup 2 [17]. Additionally, the PL population was found to have two genetic components and haplotypes from two haplogroups. In this population, 25 individuals were collected from *Cam. meiocarpa* (8)/*Cam. Oleifera* (17) plantations and their haplotypes corresponded to H8/H15, H19, and H22–H27. A probable cause is that *Cam. oleifera* planting areas have been expanding to new plantations, and the low-yield transformation for several decades has led to an increased distribution of hosts and an increase in *C. chinensis* migrants between *Cam. meiocarpa* and *Cam. oleifera* plantations. However, our analyses failed to find key haplotypes or subgroups that play a significant role in connecting different haplogroups, which was caused by the limited number of samples from *Cam. meiocarpa*/*Cam. oleifera* plantations or haploid genetic markers. The high diversity and recent demographic expansion suggest that *C. chinensis* populations will continue to expand across Jiangxi plantations. Overwintering in the soil and the development of eggs/larvae within oil-tea *Camellia* fruit both contribute to avoiding insecticides and improving survival rates in new plantations. Given the genetic diversity and population dynamics of *C. chinensis*, it is crucial to prioritize population monitoring (surveys or molecular marker-based population genetic analysis) and implement control measures (such as *Beauveria bassiana* powder) for *C. chinensis* populations in *Cam. meiocarpa* plantations and low-yield transformation plantations.

## 5. Conclusions

High diversity and recent demographic expansion of *C. chinensis* were discovered in Jiangxi *Cam. oleifera* and *Cam. meiocarpa* plantations. Two haplogroups with significant genetic divergence were detected through haplotype phylogenetic relationships and networks. Haplogroup 1, collected from *Cam. meiocarpa* plantations, had more effective migrants compared to Haplogroup 2, which mainly originated from *Cam. oleifera* plantations. The PL population from *Cam. oleifera/Cam. meiocarpa* plantations included two genetic components and haplotypes from two haplogroups. These results can improve our understanding of the dispersal of *C. chinensis* across different host plants and reveal the effect of low-yield transformation on the genetic patterns of this pest. We should pay attention to monitoring and controlling *C. chinensis* populations in *Cam. meiocarpa* plantations and low-yield transformation plantations. Our future research would focus on evaluating the influence of hosts on the adaptability of *C. chinensis* with more sampling from *Cam. meiocarpa*/*Cam. oleifera* plantations based on genomic data, including differential expression of their key detoxification genes and the biological characteristics (such as generation time and feeding preference) of this pest on different hosts.

## Figures and Tables

**Table 1:** Collecting information and nucleotide diversity indices based on *COI* of *C. chinensis* from six localities in Jiangxi.

Locality (Pop)	N/E	n/ds	Host Species	Nh	Hd	π	k	S	D (*p*)	Fs (*p*)
Ganzhou, Xingguo (GX)	26.23°/115.49°	1 male; 24 master larvae	*Cam. meiocarpa*	9	0.723	0.00125	1.353	5	0.063	−4.363
Jian, Suichuan (JS)	26.19°/114.29°	1 male; 21 master larvae	*Cam. meiocarpa*	8	0.545	0.00075	0.810	8	−2.072 *	−5.750
Pingxiang, Shangli (PS)	27.81°/113.76°	1 male; 24 master larvae	*Cam. meiocarpa*	10	0.690	0.00166	1.800	18	−2.226 **	−4.251
Pingxiang, Luxi (PL)	27.49°/114.14°	1 male; 24 master larvae	*Cam. oleifera*/ *Cam. meiocarpa*	9	0.777	0.02496	27.033	75	1.411	11.326
Jiujiang, Dean, (JD)	29.47°/115.75°	1 male; 24 master larvae	*Cam. oleifera*	5	0.693	0.00080	0.867	4	−0.491	−1.210
Jiujiang, Xiushui (JX)	28.93°/114.77°	1 male; 24 master larvae	*Cam. oleifera*	4	0.557	0.00058	0.633	3	−0.504	−0.830

Latitude (N)/Longitude (E); n: no. of samples; ds: developmental stage of samples; Nh: no. of haplotypes; Hd: haplotype diversity; π: nucleotide diversity; k: average number of nucleotide differences; S: number of polymorphic sites. Asterisk (*/**) denote the significant values (*p* < 0.05/< 0.01), and the other *p* of Tajima’s D and Fu’s Fs statistics > 0.05.

**Table 2:** AMOVA results of six *C. chinensis* populations between two haplogroups inferred from haplotype relationship.

Haplogroups	Source of Variation	d.f.	Sum of Squares	Variance Components	Percentage of Variation (%)	Fixation Indices	*p*-Value
Two haplogroups	Among haplogroups	1	1769.644	23.78056 Va	86.97	FCT = 0.870	<0.0001
	Among populations within haplogroups	4	89.833	0.80482 Vb	2.94	FSC = 0.226	<0.0001
	Within populations	141	388.740	2.75702 Vc	10.08	FST = 0.899	<0.0001
	Total	146	2248.218	27.34240			
No groups	Among populations	5	1859.478	15.07316 Va	84.54	FST = 0.845	<0.0001
	Within populations	141	388.740	2.75702 Vb	15.46		
	Total	146	2248.218	17.83018			

Va: Variance components among haplogroups; Vb: Variance components among populations; Vc: Variance components among individuals.

## Data Availability

This study’s datasets are available in the online repository. The accession numbers for the haplotype sequence data submitted to GenBank are OR976178–OR976213 for *Curculio chinensis* and OR976214, OR976215 for *Curculio davidi*.

## Appendix A

**Table A1:** Distribution of mitochondrial *COI* haplotypes in six *Curculio chinensis* populations.

Haplotype	GX	PS	JS	JX	PL	JD	Total
H1	1						
H2	1						
H3	1						
H4	3						
H5	1						
H6	2						
**H7**	1		1				2
**H8**	13	14	15		7		49
**H9**	2		1				3
H10			1				
H11			1				
H12			1				
H13			1				
H14			1				
**H15**				16	10	13	39
H16						4	
H17						2	
H18						4	
**H19**				3	1	2	6
H20				5			
H21				1			
H22					1		
H23					2		
H24					1		
H25					1		
H26					1		
H27					1		
H28		1					
H29		2					
H30		1					
H31		1					
H32		1					
H33		1					
H34		1					
H35		1					
H36		2					

Bold represents the common haplotypes. GX, Xingguo in Guizhou; PS, Shangli in Pingxiang; JS, Suichang in Jian; JX, Xiushui in Jiujiang; PL, Luxi in Pingxiang; JD, Dean in Jiujiang.

**Table A2:** The genetic distances between *Curculio chinensis* and related species in Curculionidae inferred from mitochondrial *COI* sequences (1083 bp).

Sequences	Haplogroup 1	Haplogroup 2	OR976214	KX087269	MT560591	MK654677	NC045101	NC027577	KX087330	MT232762	MW023069	NC022680	NC051548
Haplogroup 1													
Haplogroup 2	0.0563												
OR976214	0.1916	0.1992											
KX087269	0.1680	0.1765	0.1279										
MT560591	0.2225	0.2145	0.2094	0.1984									
MK654677	0.2043	0.2037	0.2175	0.2029	0.2036								
NC045101	0.2226	0.2199	0.2297	0.2317	0.1958	0.1997							
NC027577	0.2184	0.2095	0.2252	0.2140	0.2222	0.2189	0.2334						
KX087330	0.1973	0.1935	0.2058	0.1966	0.1860	0.1948	0.2066	0.2087					
MT232762	0.2072	0.1985	0.2029	0.2041	0.1851	0.2053	0.2112	0.2126	0.1923				
MW023069	0.1922	0.1936	0.1947	0.2037	0.2038	0.2179	0.2095	0.1995	0.1835	0.1933			
NC022680	0.1892	0.1952	0.2055	0.2009	0.2100	0.2117	0.2392	0.2254	0.1897	0.2005	0.1710		
NC051548	0.1993	0.2000	0.2177	0.2039	0.1865	0.2006	0.2339	0.2175	0.1875	0.1886	0.2003	0.2027	

Mitogenome information used in this study: *Curculio davidi* (OR976214), *Curculio elephas* (KX087269), *Elaeidobius kamerunicus* (MT560591), *Anthonomus pomorum* (MK654677), *Ceutorhynchus obstrictus* (NC045101), *Aegorhinus superciliosus* (NC027577), *Pantoxystus rubricollis* (KX087330), *Niphades castanea* (MT232762), *Pimelocerus perforatus* (MW023069), *Hylobitelus xiaoi* (NC022680), and *Aclees cribratus* (NC051548).

**Table A3:** The population size and numbers of effective immigrants per generation between two haplogroups (Haplogroup 1 included GX, JS, and PS populations; Haplogroup 2 included JD, PL, and JX populations) using the combined mitochondrial datasets.

Haplogroups	N	Nem	
		1→	2→
1	5.18 × 10^5^	-	1.00
2	3.21 × 10^5^	**4.96**	

N: the effective population size; Nem: effective number of migrants per generation. The bold denote significant effective migrants. → denote effective number of emigration per generation.
